# Emerging Technologies for Timely Point‐of‐Care Diagnostics of Skin Cancer

**DOI:** 10.1002/gch2.202400274

**Published:** 2025-03-18

**Authors:** Jarrod L. Thomas, Adrian H. M. Heagerty, Pola Goldberg Oppenheimer

**Affiliations:** ^1^ Advanced Nanomaterials Structures and Applications Laboratories School of Chemical Engineering College of Engineering and Physical Sciences University of Birmingham Edgbaston Birmingham B15 2TT UK; ^2^ Healthcare Technologies Institute Institute of Translational Medicine Mindelsohn Way Birmingham B15 2TH UK; ^3^ Department of Dermatology University Hospitals Birmingham NHS Foundation Trust Mindelsohn Way Birmingham B15 2GW UK; ^4^ Institute of Inflammation and Ageing College of Medical and Dental Sciences Mindelsohn Way Birmingham B15 2GW UK

**Keywords:** dermatology, global healthcare technologies, point‐of‐care diagnostics, Raman spectroscopy, skin cancer, translational medicine

## Abstract

Skin cancer is a global health crisis and a leading cause of morbidity and mortality worldwide. A leading factor of malignancy remains the UV radiation, driving various biomolecular changes. With shifting population behaviors, deficiency in screening programs and reliance on self‐presentation, climate change and the ageing world populace, global incidents have been surging alarmingly. There is an urgent need for new technologies to achieve timely intervention through rapid and accurate diagnostics of skin cancer. Raman spectroscopy has been emerging as a highly promising analytical technology for diagnostic applications, poised to outpace the current costly, invasive and slow procedures, frequently hindered by varying sensitivity, specificity and lack of portability. Herein, complex and intricate progress are overviewed and consolidated across medical and engineering disciplines with a focus on the latest advances in the traditional and emerging skin cancer diagnostics. Methods detecting structural and chemical responses are categorized along with emerging chemo‐biophysical sensing techniques. Particular attention is drawn to Raman spectroscopy, as a non‐invasive, rapid and accurate sensing of molecular fingerprints in dermatological matrix with an additional focus on artificial intelligence, as a decision support tool collectively, laying the platform toward development and rapid translation of point‐of‐care diagnostic technologies for skin cancer to real‐world applications.

## Graphical abstract

With the global incidence of skin cancer on the rise and a persistent lack of rapid, noninvasive and sensitive point‐of‐care diagnostic platforms, current and emerging technologies are critically reviewed. Among these, the emerging Raman spectroscopy system is identified as a promising complementary to conventional costly and invasive methods, offering the potential to transform dermatological care on a global scale.

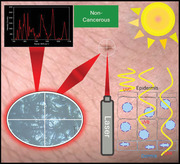

## 1 Introduction

**Figure 1:**
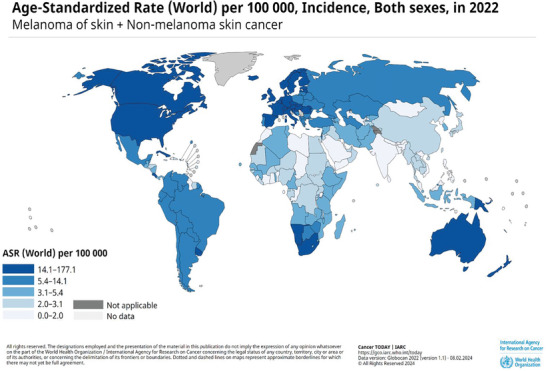
Epidemiology of skin cancer. Map of skin cancer incidence by country obtained from World Health Organization International Agency for Research on Cancer. Copyright to the World Health Organization.

Globally, skin cancer is one of the most common types of cancer with striking numbers of reported incidents, which rise each year.^[^ ^1^ ^]^ Skin cancer is typically divided into two main types, non‐melanoma (NMSC) and melanoma‐based (MSC), with the former drawing almost 2 million new cases per year internationally (**Figure** **1**).^[^ ^1^ ^]^ The true numbers are projected to be considerably higher due to the lack of statistical reporting or presentation in clinic for diagnosis. The International Agency for Research on Cancer places non‐melanoma skin cancer within the top 5 most frequently occurring cancers. Thus, notably a substantial threat to population wellbeing and health.

**Figure 2:**
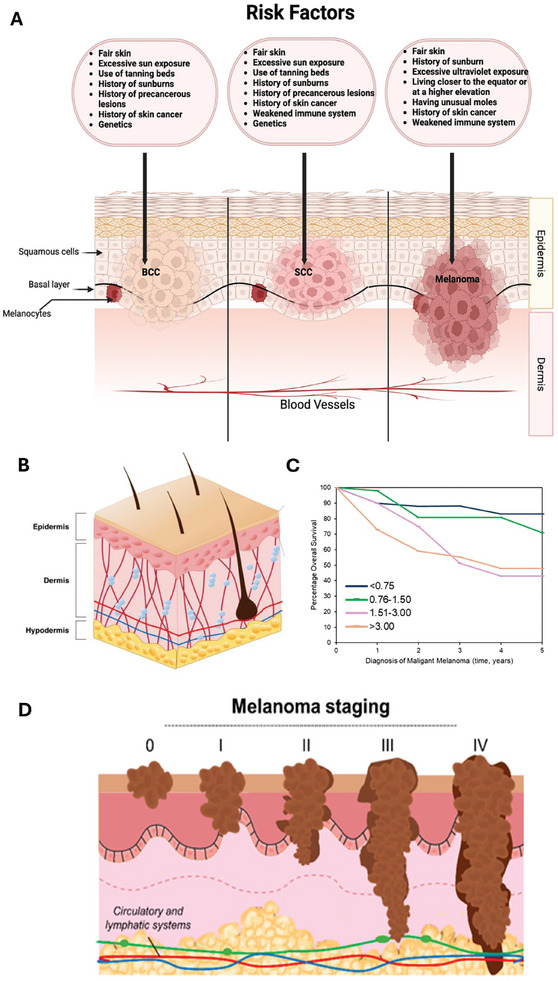
Clinical presentation of skin cancer. A) The presentation and risk factors for basal cell carcinoma, squamous cell carcinoma and melanoma reprinted from Tow et al.^[^ ^79^ ^]^ Copyright 2023, MDPI in contrast to the anatomy of healthy skin presented in B) reproduced from Brito et al.^[^ ^80^ ^]^ Copyright 2024, MDPI. C) Presents the overall survival chance (%) with time following metastatic melanoma diagnosis in years, survival analysis schematic based on initial data from Brewer et al.^[^ ^81^ ^]^ for Melanoma, dependent on grading (and in turn Breslow thickness). D) Melanoma has several disease‐specific grades/stages, from 0 to 4, and several sub‐groupings. The extent of in‐situ disease and grading relative to disease progression obtained from Matias et al.,^[^ ^82^ ^]^ Copyright 2021, MDPI with stage 4, the most advanced and of the greatest metastatic potential. Reproduced under terms of the CC‐BY license where appropriate, copyright to the authors.

NMSC (cancer arising from any skin cell other than a melanocyte) and MSC (malignancy arising from melanocytes) differ primarily on the cell types of cancer formation. Basal cell carcinoma (BCC) is a malignancy which is typically indolent (i.e., does not metastasize), originating among basal cells, a popular hypothesis is that basal cell carcinoma arises from basal keratinocyte stem cells that lie between hair follicles of the dermal‐epidermal junction and in the bulge of the hair follicle. It is hypothesized that unregulated cell growth among these stem cells leads to tumor formation. Squamous cell carcinoma (SCC), on the other hand, is the most common cutaneous malignancy outside of malignant melanoma (MM), with a propensity for metastasis. There are several types of SCC, some however, are unrelated to skin, e.g., esophageal SCC with approximately 9000 new cases yearly and a 12% 10‐year survival rate^[^ ^2^ ^]^ or squamous cell carcinoma of the lung. Actinic keratosis (AK) lesions, frequently denoted by hyperplastic patchy or scaly skin, change in color and crusty‐like appearance, and is a common precursor to SCC. If untreated, has potential to develop into a tumor.^[^ ^3^ ^]^ For cutaneous SCC (cSCC) to develop, mutations which are driven by UV‐exposure for instance must arise in long‐living cells, and these mutations typically include pyrimidine‐dimer formation. Which impact and reduce the stability of the DNA double helix.^[^ ^4^ ^]^ The stepwise progression is with mutations in p53, p16 and Ras. Melanoma skin cancer (MSC) is similarly made up of several sub‐types, with nodular melanoma the most life threatening and often diagnosed at the most advanced stages, increasing mortality rate.^[^ ^5^, ^6^ ^]^ The main types of melanomas include superficial spreading melanoma, nodular melanoma, lentigo maligna and acral lentiginous melanoma. Nonetheless, unlike both cases of BCC and SCC, mutation‐driven tumors occur in melanocytes will almost exclusively result in a degree of metastasis through the lymph nodes. Where metastatic disease is detected, most metastasize to other skin areas, respiratory system, hepatic and neurological systems.^[^ ^7^ ^]^ **Figure** **2** illustrates the structure of BCC, SCC and melanoma. The etiology of skin cancer is a complex multifactorial process which includes biochemical, biophysical and molecular changes. Typically driven by, although not exclusively, sun exposure and UV‐induced damage, with the significant mechanisms explored in depth by others.^[^ ^8^, ^9^, ^10^, ^11^ ^]^ Further drivers of skin cancer include for instance, polyoma viruses, HPV, HIV and various genetic predispositions.

Despite the continued growing prevalence of both NMSC and MSC, many countries do not carry out national screening programs. While early detection is essential in reducing morbidity and mortality caused by skin cancer, recommendations for populational wide screening programs had initially been hindered through lack of epidemiological evidence for its overall effectiveness.^[^ ^12^ ^]^ Germany, in 2008, established the World's first national screening program based on pilot data collected from some 4 years before in the SCREEN project.^[^ ^13^ ^]^ Detailed analysis of mortality had been conducted^[^ ^14^ ^]^ and the authors demonstrated initial hesitation of its overall usefulness. A further 10‐year review conducted by Hübner^[^ ^15^ ^]^ reported a marked reduction of melanoma‐associated mortality during the pilot phase, although following nationwide implementation, mortality reduction had not appeared sufficient, possibly explained by the lower powered program. The authors suggested a more targeted approach, where individuals are difficult to reach, such as personal invitations, would further drive the screening success. More generally, Del Marmol^[^ ^16^ ^]^ examined the success of the *Euromelanoma campaign*, which is a Europe‐wide public drive focused on raising the awareness of melanoma prevention and early diagnosis, taking an active position in promoting the policies at a political level. Although, largely, the campaign still encourages self‐examination and presentation, with education at a national level, healthcare‐driven screening remains non‐centralized. Since the creation of the *Euromelanoma campaign*, the majority of European countries have joined the scheme.^[^ ^16^ ^]^

The United States, via the US Preventive Services Task Force, considered screening for skin cancer in adults. In a landmark evidence report published in JAMA,^[^ ^17^ ^]^ Wernli and co‐workers, examined a general risk of skin cancer in people older than 15 years with the primary endpoints of incidence and mortality of melanoma, the accuracy of screening and the associated harms. The consensus reached determined that only limited evidence existed for skin cancer screening and that future research should be focused on the effectiveness of those considered at higher risk for skin cancer development. Much of the primary literature surveyed had a particular emphasis on melanoma screening, although not unexpectedly, as the most lethal form. With the consensus not reached on the population benefit and efficiency, continuing to be a much‐debated topic, in the absence of the national screening programs discussed above, diagnosis often relies still on patients, particularly since signs appear on sun‐exposed anatomic regions, self‐presenting to a clinic for investigation, where a typical diagnosis is based on physical examination by a clinician and subsequent biopsy. The latter, while simultaneously costly, painful and resource heavy, is considered the current diagnostic “Gold‐standard.”^[^ ^18^ ^]^ Early‐stage diagnostics are, thus, particularly challenging because of the often‐subtle epidermal differences and either none or non‐specific symptomatology, e.g., dermatitis. The development of contemporary diagnostics to replace traditional histology routes, irrespective of pathology stage are further complicated by the often‐subjective analysis of biopsies, and where pathologist detection sensitivity and specificity is dependent on a variety of factors including training, experience and resources.

The advancement of investigative techniques, such as machine learning, are confounded by the achievable highest levels of sensitivity and specificity by the current gold‐standard only, as many of these newer platforms require training or input to be of a comparable nature. Stone et al. highlighted briefly the difficulties of the currently imperfect “gold‐standards” and specifically, the calibration of novel technologies, including the barriers to attesting greater accuracy particularly, when calibration and training were conducted on already flawed processes.^[^ ^19^ ^]^

## 2 Overview of Skin Structure and Areas of Investigation

Comparative to other organs, the skin is by far the largest of the body with an estimated size of 2 m^2^.^[^ ^8^ ^]^ It is complex in cellular composition consisting of three principal constituents of epidermis, dermis and subcutaneous tissue. The uppermost layer, the epidermis, is formed from sub‐layers, namely the stratum corneum, stratum lucidum, stratum granulosum, stratum spinosum and basement membrane.^[^ ^20^ ^]^ Below this is the dermis, which is composed of two sub‐layers: the upper of the two, papillary dermis, while the lower is reticular dermis. The former is considerably thinner and centered on fibroblasts, general vasculature (blood vessels etc.), collagen and immune cells. The papillary layer also includes adipose tissue and neurons,^[^ ^21^ ^]^ as opposed to the latter, reticular dermis, which is characterized as a thick layer containing similar vasculature, in addition to adipose cells, elastin and collagen.^[^ ^22^ ^]^ Throughout the two sub‐layers, a gelatinous material, amorphous in nature, and referred to as ground substance is present as part of the extracellular matrix, having active roles in metabolism and energetics, among other functions such as tissue proliferation and development.^[^ ^23^ ^]^ This ground substance is influenced by a variety of factors such as, blood calcium concentration^[^ ^24^ ^]^ and inflammation.^[^ ^25^ ^]^ Thus, this suggests ground substance possibly has an important role in the diagnosis of neoplastic and other dermatological disease. Finally, the subcutaneous tissue, also referred to as hypodermis, is predominantly made up of adipose and connective tissues, tasked with structural support.^[^ ^26^ ^]^ With the already unquestionably complex structure of skin, there are further intricate features to each of these layers as they are made up of a series of cells and other sub‐structures. The most common cell type of the epidermis is the keratinocyte, although, as demonstrated by Rheinwald and Green,^[^ ^27^ ^]^ keratinocyte formation required fibroblast presence—at least in culture. Another differentiated keratinocyte, the corneocyte, are organelle‐free terminally differentiated keratin‐dominated desmosome‐linked cells constructing the stratum corneum, used to form part of the lipid barrier.^[^ ^28^ ^]^ The epidermal basal layer contains, in addition to the usual keratinocytes, Merkel cells and melanocytes, the latter responsible for melanin, which causes pigmentation and is released from melanosomes. Merkel cells are in control of the sensation of touch and signaling and are positioned along the dermoepidermal junction.^[^ ^29^, ^30^ ^]^ Additionally, from each of these cell types and sub‐cell types, a variety of proteins, e.g., keratohyalin are produced by granules from keratinocytes of the stratum granulosum.^[^ ^8^ ^]^ Many other cells types differentiate to form glands and skin appendages, and these are reviewed elsewhere by Tobin and Hwa et al.^[^ ^8^, ^31^ ^]^

## 3 Cancer Related Changes in Skin

With the heightened interest in markers for the early detection of disease, including cancer, specific discovery of biochemical and physical indicators are contemporary issue for many researchers. Cancer pathology and the progression of disease is often complex and results in shifts in several biomolecules, such as epigenetic modifications, fundamental genetic changes (RNA/DNA), amino acids, proteins, lipids, and overall metabolic pathway remodeling (e.g., Warburg effect). Herein, we explore some of these changes to illustrate not only the complexity of cancer progression but also to highlight potential candidate targets for utilization in the diagnosis of a variety of skin cancers and other dermatological diseases.

**Figure 3:**
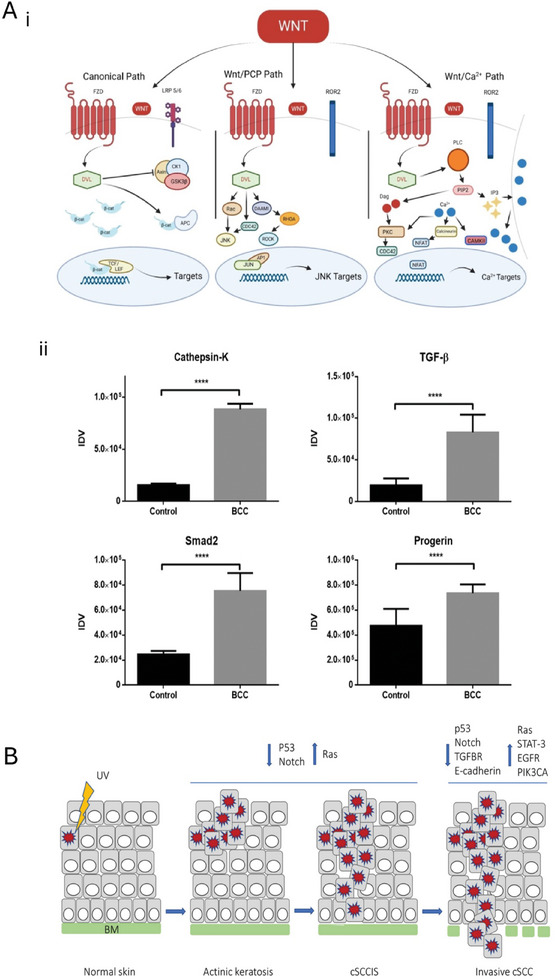
Molecular mechanisms of skin cancer. A‐i) A selection of signaling pathways and target genes which are impacted during the development of skin cancer, reprinted from Di Bartolomeo^[^ ^100^ ^]^ Copyright 2023, MDPI and (ii) the overexpression of cathepsin‐K, TGF‐β, Smad2 and progerin (adopted with permission.^[^ ^35^ ^]^) Copyright 2018, Spandidos Publications (Oncology Letters) Permission obtained directly from publisher. B) Demonstrates the transition from normal skin to actinic keratosis to cutaneous squamous cell carcinoma in situ to invasive cutaneous squamous cell carcinoma, with notable increases in Ras signaling and reductions in p53 and Notch for the initial change from actinic keratosis to in situ cSCC, for invasive and metastatic cSCC, further downregulations of TGFβ‐receptor and E‐cadherin are reported, while upregulations of STAT‐3, EGFR and PIK3CA were reported. TGF‐β: Transforming growth factor beta; STAT‐3: Signal transducer and activator of transcription 3; EGFR: epidermal growth factor receptor; and PIK3CA: phosphatidylinositol‐4,5‐bisphosphate 3‐kinase catalytic subunit 3 [also known as p110‐α]; B) Reproduced with permission.^[^ ^46^ ^]^ Copyright 2021, MDPI. Appropriate figures used under terms of the CC‐BY license, copyright to the authors.

To provide context to the caliber of protein‐based change, research published by Qendro et al.,^[^ ^32^ ^]^ initially using 5 cell lines of melanoma, processed by tandem mass spectrometry, resulted in over 4750 identified proteins. Of these, some 200–300 were differentially expressed in each cell line. Literature overview provides an indication of several possible targets, some albeit more fruitful, and individually verified by other researchers on separate occasions. These include the polycomb proteins, e.g., BMI‐1 or SUZ12, and EZH2 (Enhancer of Zeste Homolog 2), all reported in most skin cancers inclusive of melanoma.^[^ ^33^, ^34^ ^]^ From Qendro and and co‐workers research, they confirm histone H2A1B, annexin A1, vimentin, nestin, dipeptidyl peptidase IV and fibronectin as promising targets for the differentiation and diagnosis of melanoma‐based malignancies. Further, Penta et al. observed increases in Bcl‐2 and Bcl‐xl, while reporting decreases in Bax, Bim, Bak, Bid, Apaf1 and caspase‐3.^[^ ^34^ ^]^ Of the research dedicated to the detection of BCC and SCC pathologies, increases in transforming growth factor beta (TGF‐β), small mother against decapentaplegic homolog 2 (SMAD2), cathepsin‐K, progerin and matrix metallopeptidase (MMP‐1), ‐3, ‐8 and ‐9 were all found in cutaneous BCC by Ciążyńska et al.,^[^ ^35^ ^]^ **Figure** **3**. Whereas in a murine model of SCC, Zanivan et al.^[^ ^36^ ^]^ reported decreases in both desmosomal proteins “Dsg1” and “Dsg2,” and with desmosomes’ role centered on mechanical junctions being indicative of disintegration and breakdown of cell interaction. Zanivan and co‐workers also noted decreases in keratin‐based proteins (i.e., Krt1 and krt10). Further work showed evidence to suggest vasodilator‐stimulated phosphoprotein (VASP) and FSCN1 are required for cell invasion, as silencing of these led to a marked reduction in pathology. While imperative to obtain biomarkers that detect presence of disease, it is equally important to provide markers that indicate disease progression or resolution. Zanivan et al. reported PAK group of proteins, namely PAK 1, 2 and 4, all sharing increased phosphorylation states when tumors progress toward the latter disease stages. Similarly, work by Eberle et al.^[^ ^37^ ^]^ illustrated a role for tumor necrosis factor (TNF)‐related apoptosis‐inducing ligand (TRAIL) and FAS ligands in pathology. Beyer et al. in their review also concluded the presence of TNF as a clear mark of tumor progression.^[^ ^38^ ^]^ The full extent of protein involvement has been previously reviewed,^[^ ^10^, ^39^, ^40^ ^]^ and herein we summarize the significant potential for diagnosis at the protein‐level. With BCC so abundantly frequent in the population and treatment often more somewhat clinically accessible, BCC is arguably the subject of less intense research efforts compared to melanoma and cSCC. Some established articles and more recent works have focused on the influence and impact of the more pivotal role of the hedgehog signaling pathway. Chmiel et al. examined in their 2022 review the most prominent hedgehog signaling molecules in the development of BCC, namely patched homologue 1 (PTCH1) and smoothened homologue (SMO).^[^ ^41^ ^]^ In addition to these, other more notorious changes occur with p16 and p53, for example, Conscience et al. in a comparative study found not unsurprisingly overexpression of p16 in cutaneous carcinomas predominantly located in sun‐accessible skin.^[^ ^42^ ^]^ The study suggested UV‐exposure was associated with p16 induction and subsequent overexpression. This latter association echoed by others more widely, whether through earlier reviews of cutaneous tumorigenesis mechanisms by Hussein^[^ ^43^ ^]^ or primary investigations, e.g., Eshkoor et al. and Pavey et al.^[^ ^44^, ^45^ ^]^ The role of p53 in the development and pathogenesis of skin cancer is not to be underestimated. It is one of the more significantly identified contributors to cancer development systematically and is not exclusively associated with skin cancer. Albeit a significant player in the progression of cSCC,^[^ ^46^, ^47^, ^48^ ^]^ p53 was considered guardian by Ziegler and co‐workers in their 1994 Nature letter, in which they further considered UV‐exposure to act as not only the initiator of tumors but simultaneously the promotor. More comprehensive accounts of p53's role in the context of skin tumorigenesis was written by Benjamin and Ananthaswamy^[^ ^49^ ^]^ and more widely by Kastenhuber and Lowe^[^ ^50^ ^]^ and Kruse and Gu.^[^ ^51^ ^]^

**Figure 4:**
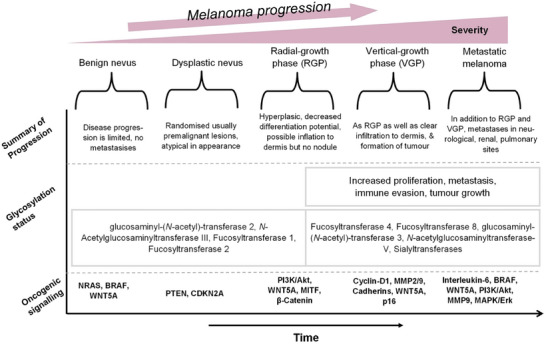
Molecular mechanisms of melanoma. A Potential progression map of a nevus to primary melanoma and metastatic melanoma over time. Progressing oncogenic signaling and glycosylation status. NRAS: neuroblastoma RAS viral oncogene homolog, BRAF: proto‐oncogene B‐Raf, WNT5A: hWnt family member 6A, PTEN: Phosphatase and tensin homolog, CDKN2A: cyclin‐dependent kinase inhibitor 2A (or p16), PI3K/Akt: phosphoinositide‐3‐kinase/protein kinase B, MITF: Microphthalmia‐associated transcription factor, MMP2/9: metalloproteinases 2 and 9, MAPK/Erk (signaling pathway). Original figure generated from the works of Pacheco et al., Prasad et al., Puglisi et al., De Vellis et al. and Miller and Mihm.^[^ ^108^, ^109^, ^110^, ^111^, ^112^ ^]^

Recently, Nikolouzakis et al. provided interesting insights into other changes occurring at the higher molecular level, including telomere length, telomerase activity and/or the epigenetic markers mentioned formerly.^[^ ^52^ ^]^ Some of the involved genes have active roles in the deregulation of the cell cycle, e.g., CDKN2A, activation of proto‐oncogenes, e.g., MYCL2, or silencing of genes used to suppress tumor formation, e.g., p14‐ARK, DUSP2 *etc*., through altered methylation status (**Figure** **4**). The authors have further summarized the intimate role of microRNAs in diagnosis, therapy and prognosis as well as discussed, the emerging biomarkers requiring evaluation and validation.

Since proteins have exhibited great variation between diseased and apparent healthy tissue, it is not unsurprising that changes also exist, abundantly, in amino acid composition, such as those observed by Silveira Jr et al. in their model study for the diagnosis of BCC and melanoma.^[^ ^53^ ^]^ Measurable species include cysteine and glycine, leucine, tryptophan and proline amongst others. Some of these are further endorsed by Lieber et al. highlighting the use of Raman micro‐spectroscopy for diagnosis of skin cancers, where the authors demonstrated tryptophan was present in higher concentrations in SCC and BCC compared to normal tissue, and similarly with changes in tyrosine, proline, phenylalanine and metabolites such as glucose and glycogen.^[^ ^54^ ^]^ Specifically, the latter aligns with a broad understanding that cancer development often results in metabolic remodeling and upregulation in some certain processes, namely the Warburg effect.^[^ ^55^, ^56^ ^]^ Through these increases in lactate production (assisting with invasion of immune surveillance), increases in proteases such as kallikrein‐related peptidase 6 (KLK6), which has been implicated in early tumorigenesis and promotion of localized skin inflammation have been seen.^[^ ^57^ ^]^ Combined, these simultaneously contribute to the initiation and sustained pathological dysregulation observed and often coupled with further changes in lipids and fatty acid metabolites. Pathological changes in lipids and fatty acid remodeling are extensively reviewed by Pellerin et al. for melanoma and likewise by Wang et al., more generally.^[^ ^58^, ^59^ ^]^ The latter review also explores the therapeutic options for the improvement of treatment efficiency. Despite newer literature, research pre‐2000 had already established lipid composition in skin was a critical factor in the development of dermatological disease. For instance, Vural et al.^[^ ^60^ ^]^ observed significant increases in lipid fractions, such as triglycerides and phospholipids, and these may have roles as possible biomarkers. Some of the most significant biomarkers and indeed most studied included collagen, elastin, triolein, keratin, ceramide, melanin, composition of water and indeed the biophysical and morphological changes observed in the nucleus.^[^ ^53^, ^61^, ^62^, ^63^, ^64^ ^]^ Silveira Jr et al. recounted the critical function of actin in growth and organization of carcinoma cells and most explicitly, the expression as a marker of cancer invasiveness.^[^ ^53^ ^]^ Fendel and Schrader and Feng et al. observed increases in melanin in cases of malignant melanoma and pigmented lesions, and thus rendering these both as indicators of disease as well as discriminators from dysplastic changes or other pathological presentations (e.g., BCC and SCC).^[^ ^61^, ^62^ ^]^ Studies performed by Feng et al. demonstrated decreases in collagen content across BCC, SCC and AK, but an increase in those with pigmented lesions (PL).^[^ ^62^, ^63^ ^]^ Malignant melanoma sample changes remained absent. Similarly with elastin, there were no measurable differences in samples of MM, but an increase was observed in BCC and a decreased concentration across PL. In the instances of triolein, Feng et al. reported a decrease globally across all pathologies. Nuclear content was over double that of normal tissues in cases of BCC and increased in AK, however, there were minimal differences across SCC, PL and MM. SCC was the only incidence where keratin varied compared to normal tissue and thus, demonstrated its useability as a good discriminator between cases of BCC and SCC. Ceramide change was minimal across all pathologies apart from MM, where content had almost doubled. Not unexpectedly, melanin concentration did not vary between normal tissue and BCC, AK or SCC, which was notably different in pigmented presentation, i.e., MM and PL. Finally, water content was seen to be different only in cases of SCC and where changes were over double that compared to normal tissue and other pathologies.

**Figure 5:**
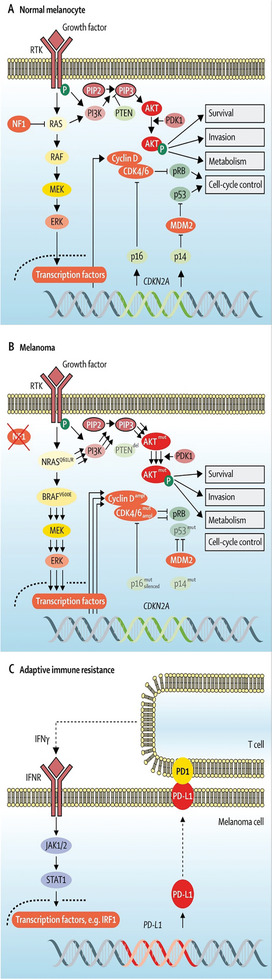
Molecular and signaling changes between a normal melanocyte and a melanocytic melanocyte. The changes in signaling pathways, growth and transcription factors associated with melanoma development in contrast to a normal melanocyte, figure adapted from Schadendorf et al.^[^ ^113^ ^]^ Copyright 2018, The Lancet ‐ Elsevier.

Many of the major biochemical groups have been considered, albeit briefly and this is not intended to be an exhaustive list of chemicals. Researchers should consider the presence of other biochemical species which could be indicative of disease, such as aromatic hydrocarbons explored by Straif et al.^[^ ^65^ ^]^ An overview diagram in Figures 4 and **5** illustrates the development and relationship of cellular signaling, glycosylation and transferases contributing to the formation of melanoma. Many of the most promising candidates for cancer detection, non‐invasively, remain at the proteomic (e.g., collagen and elastin etc.) and metabolomic (e.g., tryptophan, phenylalanine, proline, ceramides and fatty acid metabolites) levels.

## 4 Current Detection Techniques for Skin Cancer Diagnostics

Currently, the most common hospital‐based techniques for skin cancer diagnosis include dermatoscopy, optical coherence tomography (OCT), reflectance confocal microscopy (RCM), multispectral imaging and whole‐body 3D scanning, e.g., Vectra WB360. Dermatoscopy, simplistically, is a magnifying lens with a built‐in light based on transillumination in contrast to the directly applied light due the overpowering reflection generated by the stratum corneum without polarization. It has been suggested that an incident light shone on skin can result in absorption, refraction, diffraction or reflection.^[^ ^66^ ^]^ For instance, Sonthalia et al. observed reflection from skin, which is scaly and dry.^[^ ^67^ ^]^ By using polarized light or the addition of a thin layer of oil, light is reflected deeper into the skin, providing insight into deeper skin structures such as, papillary dermis and dermoepidermal junctions not visible by the naked eye.^[^ ^68^, ^69^ ^]^ While the polarized Dermascope, exploring the above imaging is inexpensive, this technique requires highly trained personnel with further detected specificity and sensitivity being dependent on experience and training.^[^ ^68^ ^]^ OCT, similarly, to the dermatoscope, is widely employed in the clinical environment and is based on a near infrared‐red light source and low‐coherence interferometry. Combining OCT with a dermatoscope enables the detection of backscattered light waves, and when analyzed with a statistic reference signal facilitates real‐time cross‐sectional imaging of the skin. This imaging depth exceeds that of the multispectral imaging (MSI), although not without its own clinical challenges (**Table** **1**). MSI systems are based on reflected or transmitted light from skin, producing spectral cubes which contain both the spatial and structural data. The spectral properties of skin are reflected as chromophores, i.e., coloring‐emitting organic molecules, known to differ between disease states relative to healthy cohorts.^[^ ^70^ ^]^ MSI however, is not the most broadly used or studied analytical tool for in vivo skin diagnosis perhaps because of the frequent issues with spectral irregularities and reproducibility.^[^ ^71^ ^]^

**Table 1:** Comparison of current and established hospital‐based methods for skin cancer diagnostics.

Technology/ technique	Advantages/benefits	Disadvantages/drawbacks	References
Biopsy	Considered as “gold‐standard” High sensitivity and specificity Allows morphological and histopathology analysis Enables histochemistry and biochemical analysis	Resource heavy (reagents & disposables) Requires specialist staff (histologist/ clinical scientist) Costly Psychologically challenging for patients coupled with long result waits	[18, 90]
Dermatoscopy	Cost‐effective Results/outcome instantly available Visualize structures not detectable to the eye, i.e., papillary dermis and dermo epidermal junctions	Requires interpretation and the examination by a clinician varies in sensitivity and specificity dependent on physician training Technique useful only to trained dermatologists	[68, 69]
Optical Coherence Tomography (OCT)	Allows extended imaging depth compared to that of reflectance confocal microscopy Is a non‐invasive technique and intra‐operative use during Mohs surgery has been observed to guide surgeons during margins assessment	Poor imaging resolution Size of the machine Requirement for specialist training difficulty of pin‐pointing the exact location being scanned	[91, 92]
Reflective confocal microscopy (RCM)	Permits the examination to the depth of the dermal‐epidermal junction The technique coupled with artificial intelligence (AI) can improve specificity of detection, reporting a ROC >80% for BCC for example	Analysis of RCM images requires extensive experience and training Early career clinicians often lacked agreement or consistency among diagnosis, as a result the technology sees a lower diagnostic accuracy compared to others Not portable, bulky	[93, 94, 95]
Whole body 3D imaging	It is possible to monitor the total surface area for lesions and pigmentation through a single scan, which can be followed up over several years to track longitudinal changes Allows incorporation with further tools such as DermaGraphix Loupe Tool, adding additional modalities for superior assessment of the Canfield Vectra WB360 system for example. Allows integration with AI and machine learning to improve recognition and sensitivity of analysis	Exceedingly large size/ comparable or larger than CT scanner Expensive to purchase and maintain	[96]
Self‐mixing interferometry (SMI)	Contactless, compact and low energy consumer Can self‐align to lesion and is possible to incorporate this technology with other modalities, e.g., confocal microscopy to enable distinguishing of normal and high vascularized tissue in the case of tumor angiogenesis for example.	Increases in uncertainty as the focal point penetrate tissue layers, possibly missing abnormal cells.	[70]
Multispectral imaging	Transformation of black and white and RGB images to higher color dimensions For LED‐systems, technology is low cost, usually small, energy efficient. Variable but acceptable sensitivity/ specificity	Time‐consuming Cumbersome Requires complex system (mechanical) configuration Poor spatial and spectral resolution	[70]

## 5 Increasing Focus of Artificial Intelligence for Dermatological Diagnosis

The official definition, according to the Oxford English Dictionary of artificial intelligence (AI) is – “the capacity of computers or other machines to exhibit or simulate intelligent behaviour; the field of study concerned with this.”^[^ ^72^ ^]^ While AI use is established in some clinical practice, for several specialties including dermatology, integration and use are comparatively novel and where offered, frequently limited and primitive. With rising interest in AI and machine learning, for instance the contemporary and ongoing awareness of ChatGPT,^[^ ^73^, ^74^, ^75^ ^]^ simply, expectation will see clinical AI usage increase across specialties,^[^ ^76^, ^77^ ^]^ despite much caution and resistance to clinical transformation.^[^ ^77^ ^]^ Nevertheless, some technologies have funneled through to perspective clinical use, namely the VECTRA WB360 system, which makes use of machine learning, a sub‐type of artificial intelligence, with the capability to detect, analyze and classify lesions of interest. For instance, one artificial intelligence‐based technology for clinic use is highlighted via a clinical trial protocol, published by Felmingham et al.,^[^ ^78^ ^]^ ID: NCT04040114, which aimed to explore the use of convoluted neural networks in diagnosis of skin diseases, in the real‐world clinical environment. The intention was to replicate the remarkably accurate detection capabilities observed in an experimental setting. Total trial recruitment saw 214 patients enrolled with a lesion count of 743. As an early trial, the study was well powered with the primary outcome to confirm the accuracy of lesion diagnosis, compared to that of a teledermatologist.

The results reported a diagnostic potential (as area under the curve (AUC)) of the convolutional neural network to be 0.837 [95%Cl: 0.813‐0.860] compared to the teledermatologist of 0.807 [95%Cl: 0.779‐0.835] with overall *p*‐value of 0.05, suggesting tentatively being statistically significant. False negative rates varied between network and dermatologists, although importantly the authors acknowledged the convolutional neural network led to an increase in unnecessary biopsies, while other patients opted for the preferred biopsy route following the network results,^[^ ^83^ ^]^ highlighting the current landscape and distrust in artificial intelligence use within the clinic, particularly from the patient perspective. We subsequently, conducted a wider search on AI techniques for the detection of dermatological diseases and in most cases, studies saw machine learning outperform, some but not all, clinicians.^[^ ^84^, ^85^ ^]^ Further, research conducted by a University of Nottingham research team, Jones et al. systematically reviewed AI and machine learning algorithms for primary and community care environments, identifying several interesting points and a much‐needed methodological checklist with the aim of standardizing algorithm development for detection of skin cancer.^[^ ^86^ ^]^ Current lack of standardization coupled often with the absence of true patient demographics only hinder as opposed to facilitate the development of AI for point‐of‐care diagnostics.

Nonetheless, research is currently focused on the development of AI for the clinical environment, which undoubtedly has exceptional and important potential. However, there are a range of somewhat controversial questions often associated with this including, the ethical use of AI and machine learning in the clinic; who is accountable for decisions made by an AI device? What protections are in place for a clinician who uses AI in practice etc.? The usability of training data to develop algorithms and the dignity of patients among many other questions were recently reviewed expertly by Zhang and Zhang.^[^ ^87^ ^]^

RCM is considered as one of the very few techniques with the required resolution, closely similar to the histological outputs. RCM uses a diode laser as an incident coherent and monochromatic light source, which is focused on a micrometer area with the reflected light being analyzed, enabling different structures with different indices of refraction to be identified, e.g., melanosomes or membranes.^[^ ^88^ ^]^ The experimental details of the RCM are reviewed by Nehal et al.^[^ ^89^ ^]^ The overall advantages and disadvantages of the above techniques are summarized in Table 1.

## 6 Raman Spectroscopy

**Figure 6:**
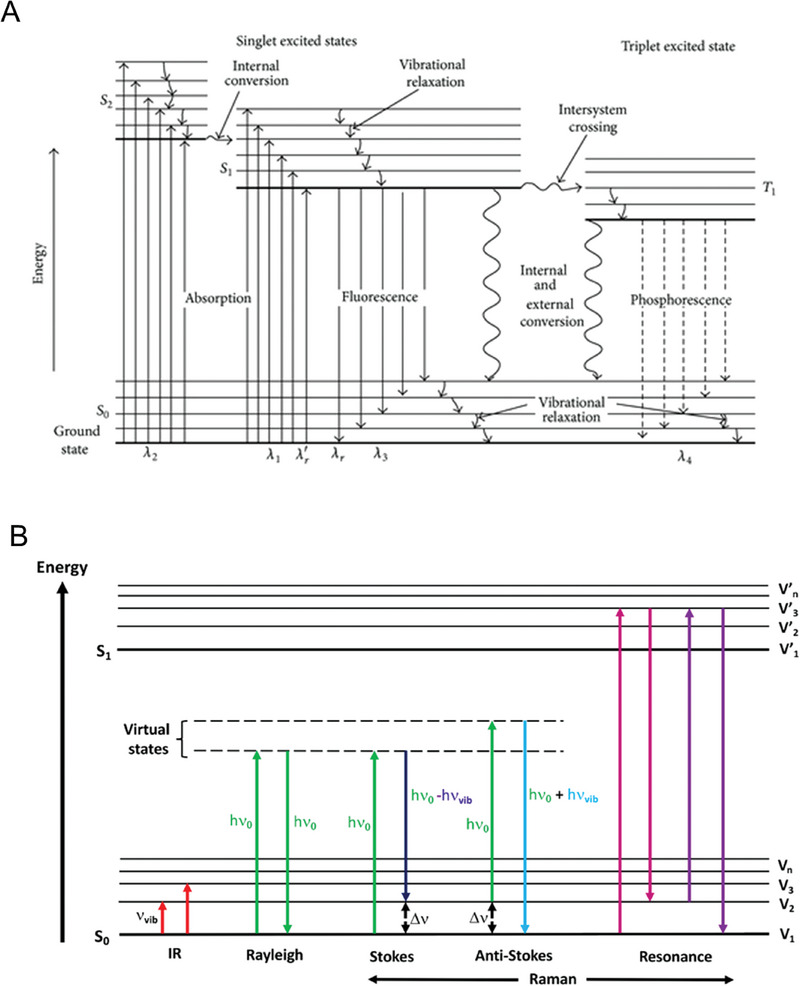
A) Jablonski diagram illustrating electronic states, vibrational relaxation and features of absorption, fluorescence, phosphorescence and system conversion used from.^[^ ^115^ ^]^ Copyright 2018, Wiley. B) Energy level changes in response to the incidence light. In Rayleigh scattering, the incident photon returns to the initial energy level (ground state), whereas in Stokes scattering it is at a higher vibrational state, figure reprinted from Geraldes.^[^ ^116^ ^]^ Copyright 2020, MDPI.

All light, including that of infrared, is regarded as electromagnetic radiation comprising of interchanging electric and magnetic fields, described classically by a continuous sinusoidal wave, characterized by features of amplitude, wavelength and phase constant. During light emission, where radiation is discharged as photons, during absorption where electrons increase in energy or during emission where there is a reduction in electron energy with emittance of a photon, an electronic state change is observed, frequently demonstrated via a Jablonski diagram (**Figure** **6**A). When electromagnetic radiation strikes non‐homogeneity matter (or a scattering material, e.g., liquids, gases or solids), light is either scattered or reflected. During this interaction, oscillation of the electron cloud drives temporary or sporadic charge separation, commonly referred to as an induced dipole moment^[^ ^97^ ^]^ This induced dipole moment can result in light scattering with two principal phenomena. If the photon energy remains unchanged, this can result in a type of elastic scattering: Rayleigh scattering, Thomson or Mie scattering. Thomson scattering is centered on electron‐photon, with the primary use in high‐energy X‐ray for medical detection.^[^ ^98^ ^]^

The other scattering phenomenon, and the most significant in Raman spectroscopy is known as Raman scattering, which is an inelastic scattering type. During this process the photon energy is not conserved. Instead, if incident light is greater than that of the scattered light, Stokes lines are observed, as opposed to where the incident light is less than that of scattered light, anti‐stokes lines are notable (Figure 6B). A typical Raman spectrum consists of Rayleigh lines, which are of a greater intensity compared to Stokes and Anti‐stokes, as a result of elastic scattering being the most frequently occurring of both, and which only observed in approximately 1% of the time.^[^ ^99^ ^]^

**Figure 7:**
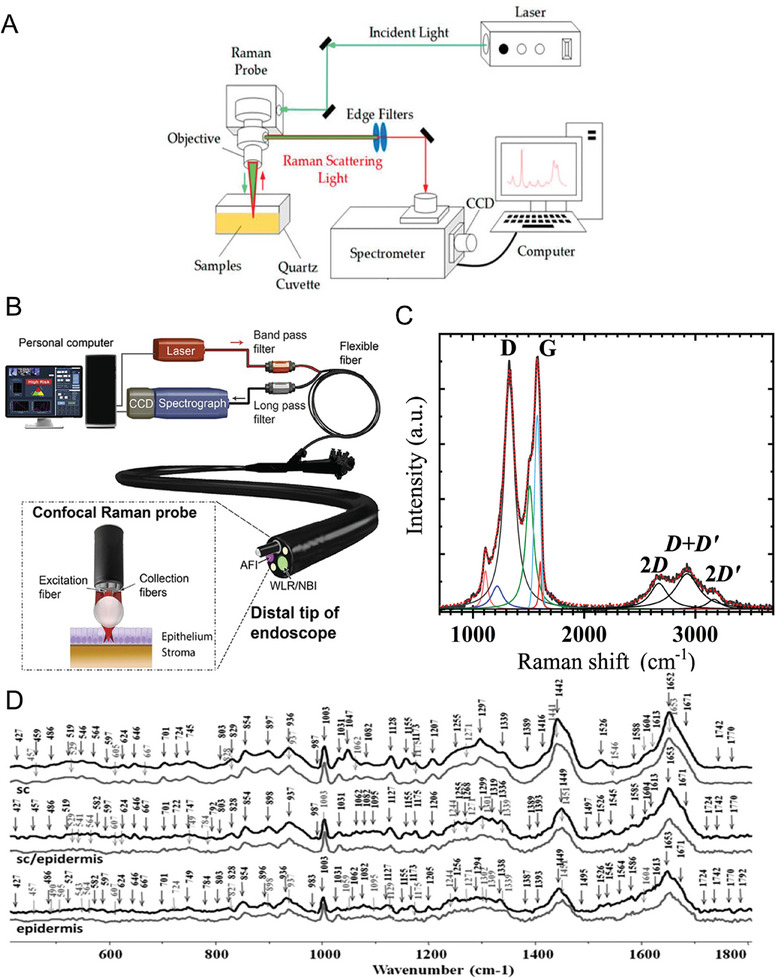
Raman systems and spectral output. A) A schematic diagram of a general Raman spectroscopy system used with permissions from.^[^ ^121^ ^]^ Copyright 2017, MDPI. B) An example of a clinical fiber‐optic based system demonstrated by Heng et al. and obtained from.^[^ ^122^ ^]^ Copyright 2020, Wiley. Representative Raman spectra of C) graphene with characteristic peaks reprinted from Pinto et al.^[^ ^123^ ^]^ Copyright 2021, MDPI and D) tentative peak assignments of pigskin (gray line) and human skin (black) demonstrating the inherently more complex spectral features, obtained from Tfaili et al.^[^ ^104^ ^]^ Copyright 2012, Analyst (RSC). Appropriate figures used under terms of the CC‐BY license, copyright to the authors.

A typical Raman system consists of a laser, which may include a diode laser, helium‐neon, argon or Ti: Sapphire for instance, a range of specific lenses, filters, mirrors and dichroic mirrors (which have differing transmission and reflective features), a microscope, objective and a sample holder (**Figure** **7**A). Other systems, such as fiber‐optics based, make use of fiber‐optic cables to excite the sample and transmit the signal (Figure 7B). In addition to the optical components, systems also typically include a central controller (e.g., PC), and a detector for acquisition of signal, such as charge‐coupled device.^[^ ^101^ ^]^ Signal obtained through the charge‐coupled device spectrometer is managed by the central controller and plotted as a spectrum, which is the product of intensity of scattered light represented as counts on the *y‐*axis and the frequency of light on the *x*‐axis, which is typically denoted as a wavenumber or interchangeably as Raman shift per centimeter.

From a spectrum, several key pieces of information are obtainable, namely the homogeneity or lack of from spectral position, concentration through intensity calibration curves, substance stress state through individual band changes and importantly, the identification of a substance through combined wavenumber shifts and intensity. The positional location of a shift‐based peak is dependent for instance, on atomic mass of the bond, where the larger atomic masses result in lower Raman shifts in contrast to the lower masses yielding higher spectral shifts.^[^ ^102^ ^]^ For instance, for graphene (Figure 7C), there are three dominant peaks including, the “G‐band,” “2D‐band” and “D‐band.” The sharp peak at 1585 cm^−1^ is primarily due to in‐plane stretching of sp^2^‐bonded carbon atoms, whereas the D‐band is associated with the peak preceding the G‐band and similarly associated with sp^2^‐bonded carbon, and more specifically, the breathing mode of carbon rings. Finally, the 2‐D band of graphene is often used to specify the number of layers in the studied sample.^[^ ^103^ ^]^ Depending on the structure, composition or species under investigation, the complexity of spectra differs. As observed for graphene, only few peaks are notable, whereas, biological spectra, such as those obtained from skin are inherently more intricate and considerably more complex (Figure 7D), due to the presence of a variety of biomolecules, e.g., proteins, lipids, amino acids amongst other. For instance, Tfaili et al. identified over 60 peaks in skin samples (Figure 7D) with the most notable including the 854 cm^−1^ assigned to tyrosine and polysaccharides, the 936 cm^−1^ assigned tentatively as vibrations of amino acid side chains, stretching of C─C bond and protein *α*‐helix.^[^ ^104^ ^]^ Other characteristic peaks included the 1003, 1297 and 1442 cm^−1^ attributed to phenylalanine, nucleotides and deformation of lipid CH_2,_ and cholesterol, fatty acids as well as further deformations of CH_2_ and CH_3_, respectively. Raman shift at 1652 cm^−1^ was found to be indicative of several key molecules, including protein reassignment in elastin, stretching of carbonyl groups and lipids and amide I and amide I *α*‐helix.^[^ ^104^ ^]^ Following the identification of these peaks and the associated ratios of, for instance, 1670 cm^−1^ and 1650 cm^−1^ representative of the β‐sheets to *α*‐helix respectively, it is now possible to indicate the folding state and conformational structure of amino acids and subsequently infer protein assembly. These, when combined, can provide important insights into the potential malignancy development since the confirmational change is known to be associated with alteration in biological function and control in several key areas, including stress response, DNA damage repair and especially in the unfolded protein response, which is a critical factor in the progression of melanoma for instance, and in the contribution to neoplastic progression.^[^ ^105^, ^106^, ^107^ ^]^

## 7 Advantages and Disadvantages of Raman Spectroscopy Used in Cancer Detection

Raman spectroscopy has been used for the detection of different cancers, including skin cancer,^[^ ^114^ ^]^ however, Raman spectroscopy is not without limitation amongst its successes. While it excels as being non‐invasive and non‐destructive (caution laser power could damage tissue) compared to other methods such as biopsy or mass spectrometry, incidents of low sensitivity not necessarily observed with other destructive techniques does not go unnoticed.

Thus, particularly when coupled with the acquisition of weak signals increasing time requirement to obtain measurements and substance concentration limit of detection, confines clinical usefulness.^[^ ^117^ ^]^ Traditional objective‐based Raman spectroscopy systems, such as the one described by Wang et al.,^[^ ^118^ ^]^ have faced challenges, particularly in maintaining lens focus over extended periods, which can be problematic for patients with conditions like akathisia or attention deficit hyperactivity disorder. However, these limitations have been addressed with the development of rapid clinical fiber‐optic‐based systems, as demonstrated by Lui et al.^[^ ^114^ ^]^ Their approach utilized a rapid dispersive near‐infrared system, enabling spectral acquisition within just one second.^[^ ^119^ ^]^

However, advantageously, Raman spectroscopy enables the concurrent detection of a range of micro‐ and macromolecules with minimal sample preparation, i.e., a label and dye‐free technique and with high specificity, which is ideal for living systems where water causes little spectral noise.^[^ ^117^ ^]^ While not a limitation exclusively experienced with Raman, as similarly shared with mass spectrometry and nuclear magnetic resonance, interpretation of data as to ensure that it is biologically and clinically meaningful, requires extensive statistical analysis to identify changes not often observable without such processing. This is primarily due to the high dimensionality of data and often subtle biochemical changes. Although analysis varies between groups, it almost exclusively requires dimensionality reduction such as principal component analysis, partial‐least squares or machine learning techniques, e.g., Banbury et al.^[^ ^120^ ^]^ One of the drawbacks of Raman spectroscopy, typically, is that biological samples are often prone to autofluorescence,^[^ ^117^ ^]^ making detecting and analyzing the output signal challenging, particularly for fluorophores such as melanin. Nevertheless, using a different laser wavelength can significantly reduce fluorescence from the studied biological samples.

In addition to the summarized and overviewed substantial technology‐based challenges for detection of cancer in the clinic, further difficulties exist chiefly at the regulatory level. As far as we are aware, no Raman‐based clinical device has been authorized for sole use in diagnostics by the Medicines and Healthcare Products Regulatory Agency for the UK healthcare services. Device approval is a long and notoriously complex affair. This challenge is further compounded by the healthcare competent authority in the United States, Food and Drug Administration (FDA), which has not yet authorized Raman use in the US healthcare facilities albeit recently, the FDA has granted authorization for Raman spectroscopy to be used for content uniformity analysis for medicinal and drug products. Overall, to date, despite the many emerging diagnostic developments for disease diagnostics and minorizing^[^ ^124^, ^125^ ^]^ Raman technology still lacks standardization and regulatory approval as a method across the medical field, despite being a highly promising technique, which inhibits the translational pathways to the clinical environments.

## 8 Early Clinical Studies of Raman Spectroscopy in the Diagnosis of Skin Cancer

Interest in Raman spectroscopy for cancer diagnostics has been of a growing interest recently, a statement which is supported by the increasing number of publications over the last decade. Generally, many of the initial publications provided the foundation to differentiate healthy and diseased tissue, for instance by Gniadecka et al. who identified profound changes in amide I and III bands in cancerous tissue (skin lesions, both benign and malignant) when compared to healthy, observations which can now be attributed to increased DNA, amino acids, and protein presence.^[^ ^126^, ^127^ ^]^ Gniadecka and co‐workers also illustrated variation in lipid and keratin concentrations and similarly, Fendel and Schrader et al. demonstrated amide and protein backbone changes, collagen and varying intensity of nucleic acid peaks.^[^ ^61^, ^126^ ^]^ Later Choi et al. suggested it was feasible to precisely differentiate healthy and diseased tissue through the identification of distinct peak profiles, many of which were between Raman shifts 1250–1350 cm^−1^.^[^ ^128^ ^]^ Several of these Raman shifts were identified as amide III, lipids, protein C‐N stretching in an *α*‐helix conformation, collagen and polynucleotide chains.^[^ ^19^ ^]^ Some of these peaks are expected to vary with pathology, notably in instances of uncontrolled and rapid cellular proliferation, a hallmark of cancer. More recent research by Silveira Jr et al. provided further insight to these biochemical changes, namely deviations in triolein, elastin, collagen and actin.^[^ ^53^ ^]^ Explicitly, and in agreement with several of the studies that followed, it was reported that Raman shifts in the region of 400–1800 cm^−1^ were the most significant with the potential to serve as biochemical and biophysical markers of disease. Feng et al. investigated changes associated with BCC, SCC, MM and further AK and benign pigmented lesions.^[^ ^62^ ^]^ Results in their study indicated clinical deviations in ceramides, triolein and melanin content in melanoma presentations. A follow‐up study saw changes in collagen and elastin, complementary to published findings by Feng et al.^[^ ^63^ ^]^ Lieber et al. reported further peaks of interests and Anastassopoulou et al. detailed the glycosylation and protein structure changes in melanoma patients, as well as variations in oxidative stress and cellular interactions between proteins and metabolites.^[^ ^54^, ^64^, ^129^ ^]^ Several authors concurrently have demonstrated the potential for differentiation and diagnosis of malignancy through Raman spectroscopy and especially in skin. The integration of these initial peaks of interest, serving as candidate biomarkers, with novel portable technology would serve as a powerful advancement in the early detection of cancer, monitoring of clinical responsiveness to treatment and in preventative early screening programs at the point‐of‐care.

## 9 Toward Portable Raman Spectroscopy Devices for Skin Cancer Diagnostics

In recent years, recognizing the potential of Raman spectroscopy as an analytical tool with many ramifications, increasing research has been focused on translating benchtop Raman to a portable technology for point‐of‐care diagnostics.^[^ ^130^, ^131^ ^]^ Portable Raman technologies have been successfully developed in several non‐medical fields including for instance, forensics,^[^ ^132^ ^]^ pharmaceutical and food analysis^[^ ^133^ ^]^ contributing towards early but growing attempts for point‐of‐care (PoC) diagnostics. Notable examples include Rickard et al. who demonstrated use of rapid optofluidic surface‐enhanced Raman scattering (SERS) devices for detection of traumatic brain injury at the PoC^[^ ^134^ ^]^ and Stickland et al. who employed a probe‐based approach to intracranial TBI monitoring.^[^ ^135^ ^]^ Raman spectroscopy has similarly been utilized for the diagnosis of Alzheimer's disease through analysis of cerebrospinal fluid, as demonstrated by Ryzhikova and co‐workers.^[^ ^136^ ^]^ The authors provided an alternative method to current diagnosis which is based frequently on, either a CT/MRI scan or clinician assessment only and thereby is limited by physician experience and training. This technology, among others, could be transformed to portable Raman systems. Other examples, which include breast cancer diagnosis^[^ ^137^ ^]^ and oral disease assessment,^[^ ^138^ ^]^ which stresses the versatility of Raman for poly‐wide specialties in a primary, secondary, and tertiary care setting. Alike those previously mentioned, Raman spectroscopy can also be utilized to differentiate diseases within each class, e.g., non‐healing and healing mucosal tissue in inflammatory bowel disease^[^ ^139^ ^]^ and for the discrimination of disease with differential diagnosis, such as ulcerative colitis and Crohn's disease in primary and secondary care.^[^ ^140^ ^]^ Use of saliva for the differentiation of these diseased states is a considerably less invasive method than the current method, which is central to endoscopic testing and imaging.

Together, disease which has the potential to be diagnosed by Raman spectroscopy, can be transformed to a portable technology and thereby be available at PoC with results available almost instantaneously. Thus, having far reaching consequences, both economically and reducing care waiting times, especially in specialties where waiting lists are notoriously long.

**Figure 8:**
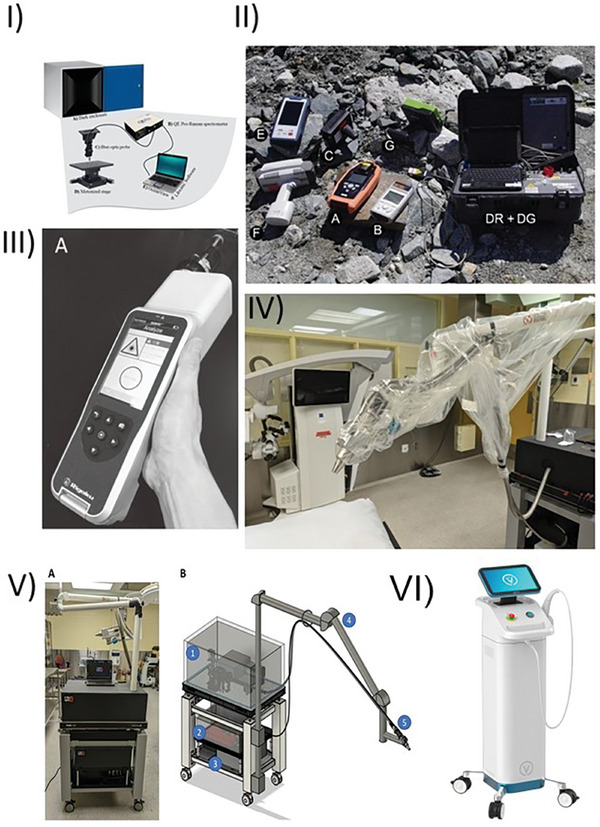
Portable Raman systems demonstrating variation in size, portability and style. I) Portable technology utilized to detect spoilage in food.^[^ ^133^ ^]^ Copyright 2022, MDPI. (II) Miniatured Raman spectrometers used to beryl in a model study a) FirstDefender XL, b) FirstDefender RM, c) Inspector Raman, dr/dg: EzRaman‐I, e) Bravo, f) FirstGuard, and g) RaPort as reviewed by Jehlička et al.^[^ ^149^ ^]^ Copyright 2017, Wiley. III) Progency spectrometer employed for the detection of colorectal cancer adapted from.^[^ ^150^ ^]^ Copyright 2019, J‐Stage. IV) Illustration of an intra‐operative system for the detection of skin cancer, showing a sterile drape secured with bands and sterile tape, while attached to a telescopic arm to aid in removability and insertion into surgical cavity (V). Both Va and Vb demonstrate overall device setup – used with permissions from.^[^ ^141^ ^]^ Copyright 2023, RSC. (VI) The most currently advanced commercial clinical Raman system by Vita Imaging Inc., used with explicit written permission,^[^ ^148^ ^]^ copyright to the authors.

In dermatology and oncology, particular examples of Raman spectroscopy include those authors’ whose primary aim was the development and validation of a portable device for effective intra‐operative cancerous margins assessment, where they demonstrated based on a trained model which reached 96% accuracy for tissue border visualization. Study findings confirmed clear differentiation of adipose tissue and muscle.^[^ ^141^ ^]^ Cautiously, the model was developed, validated and tested only on a limited number of pigs (*n *= 3), which may lack data with the natural variation that is associated and observed especially with skin, this was accounted for as much as possible with a high number of spectra acquired. Generally, Daoust and co‐workers highlighted the early successes of Raman for intra‐operative management. Former to their work, however, Khristoforova et al. provided data based on a portable (and cost effective) Raman spectroscopic system, with which they saw specificity and sensitivity range from 78.9 to 100%, and with area under the receiver operating characteristic (AUROC) greater than 0.92 for all measured parameters, which is an impressive clinical predictor of disease.^[^ ^142^ ^]^ Bratchenko et al. similarly to Khristoforova and co‐workers, saw varying AUROC success ranging from 0.69 to 0.81, which remains a diagnostic predictor greater than that of GPs in primary care and trainee clinicians, but comparable or less than that of a trained consultant dermatologist.^[^ ^143^ ^]^ Conceivably, primary care would benefit from portable Raman systems for initial diagnostic management. Lieber and Mahadevan‐Jansen, followed by Lui et al., introduced real‐time integrated prototype systems for in vivo skin diagnostics, achieving operating characteristic curve values with mean AUCs exceeding 0.860 across various skin comparisons. Independent clinical validation studies by Zhao et al.^[^ ^144^ ^]^ further supported these findings, reporting a highest AUROC of 0.894 with robust confidence intervals. Their earlier work also demonstrated similar AUROC values (e.g., 0.986), with Zhao et al.^[^ ^145^ ^]^ reporting diagnostic specificity and sensitivity ranging from 0.891 to 0.911. Collectively, these advancements, combined with multivariate analysis platforms, underscore the potential for benchtop Raman systems to transition into clinical settings as portable devices with strong diagnostic performance for skin cancer detection.^[^ ^64^, ^114^, ^146^, ^147^ ^]^ Overall, all authors have demonstrated Raman as a suitable diagnostic tool for all stages of clinical practice, which however still needs to be refined and to achieve maximum diagnostic performance, ensure highest levels of practitioner and patient safety and indeed, contribute towards the advancement of early diagnostics for optimal clinical outcomes. The technology is becoming commercially attractive with the generation of industry‐based technologies such as those developed by Vita Imaging Inc.,^[^ ^148^ ^]^ which is marketed as the most rapid and advanced system on the market for the non‐invasive detection of skin cancer. The technology is currently under review by the FDA, and a decision of outcome is expected in 2025 (**Figure** **8**vi).

## 10 Conclusions and Future Prospects

Skin cancer continues to pose a significant global health challenge, with incidence rates rising annually. Without effective prevention, diagnosis and treatment, this condition imposes an increasing burden on healthcare systems and, more critically, results in unnecessary patient morbidity and mortality. Despite the initial successes of early skin cancer screening initiatives, there is still no global consensus supporting population‐based screening programs. Consequently, routine skin cancer screening remains absent in many countries. Current diagnostic methods are often invasive, time‐intensive, costly and resource‐dependent, contributing to this gap in care. Furthermore, with symptoms predominantly appearing on sun‐exposed body areas, the onus is largely on patients to self‐present for clinical examination. Early diagnosis, however, significantly reduces treatment burden, lowers overall system costs and leads to more morbidity‐free years while decreasing mortality rates. Biopsy, the gold standard for skin cancer diagnosis, is invasive and should be reserved as a last resort, underscoring the urgent need for non‐invasive, point‐of‐care diagnostic alternatives.

This review has provided a comprehensive overview of the diagnostic systems currently employed for skin cancer detection, predominantly within secondary and tertiary healthcare settings, while presenting a unique perspective on the associated clinical and engineering challenges. It also explored the status of candidate biomarkers, highlighting the extensive cancer‐related alterations in skin, including the involvement of proteins, signaling pathways and metabolic targets. Translating these biomarkers from the laboratory to secondary care and pre‐hospital, point‐of‐care settings is progressing, with Raman spectroscopy emerging as a particularly promising technique.

This review highlights the pivotal role of emerging technologies such as Raman spectroscopy, presenting several examples of early successes in achieving timely and accurate diagnoses. The integration of Raman spectroscopy with artificial intelligence (AI) and machine learning is increasingly recognized as essential in clinical settings, offering robust support for clinicians in their role as primary care coordinators. Advances in supporting technologies, including machine learning platforms and sophisticated chemometric analysis software, further enhance the potential of Raman spectroscopy. These innovations could expand its applications to include the detection of protein abnormalities in monogenic diseases, enabling non‐invasive identification of well‐characterized protein alterations. Despite these promising developments, no commercial device has yet supplanted conventional diagnostic methods in clinical practice, primarily due to the complexities of medical device regulation and the challenges inherent in diagnosing multifaceted diseases like cancer. Nevertheless, Raman spectroscopy continues to demonstrate substantial clinical potential, not only in skin cancer diagnosis but also across a broad spectrum of applications, including emergency medicine, intensive care, neurology, psychiatry, ophthalmology and gastroenterology. Ongoing efforts are focused on developing portable Raman systems capable of performing areal scans, thereby enhancing both usability and diagnostic precision.

In summary, skin cancer diagnostics remains a dynamic and evolving field with clear objectives to enhance diagnostic speed and precision, ultimately improving patient outcomes and recovery. Advances in therapeutic interventions and diagnostic technologies will synergistically enhance the patient care continuum and reduce global mortality from this disease. Although current diagnostic methods face limitations in specificity, sensitivity and timeliness, the ongoing development of Raman spectroscopy and other innovative approaches offers significant promise. The realization of these technologies in clinical practice will undoubtedly mark a transformative step in skin cancer diagnostics in the coming years.

## Conflict of Interest

The authors declare no conflict of interest.
